# Stress CMR as a gatekeeper to complete revascularisation in STEMI patients with moderate-severe bystander disease at primary percutaneous coronary intervention

**DOI:** 10.1186/1532-429X-17-S1-O29

**Published:** 2015-02-03

**Authors:** Amardeep Ghosh Dastidar, Alexander Carpenter, Elisa McAlindon, Tom Johnson, Julian Strange, Angus K Nightingale, Andreas Baumbach, Chiara Bucciarelli-Ducci

**Affiliations:** 1 NIHR Cardiovascular Biomedical Research Unit, Bristol Heart Institute, Bristol, UK

## Background

Nearly 40% of the patients presenting with ST Elevation Myocardial Infarction (STEMI) have multivessel disease (MVD). Currently ESC and the ACC/AHA guidelines recommend revascularization of the culprit artery only. 2 recent trials (PRAMI and CVLPRIT) have shown a superiority of complete in-hospital revascularisation as compared to culprit only treatment. Although the mortality in the 2 groups in the trials were not significantly different but the composite end point was, which was mainly driven by ischaemia.

## Aim

To assess the role of stress CMR as a gatekeeper to complete revascularisation in STEMI patients with moderate to severe bystander disease, treated with Primary PCI (PPCI) of the culprit lesion.

## Methods

The study was performed from the data collected on consecutive patients who underwent PPCI between September 2011 - September 2013 at a tertiary centre in the South-West of England. A non-culprit lesion was considered to be moderate to severe if the stenosis was 50-75% in large proximal epicardial vessel or 70-90% elsewhere. Severe or critical bystander disease was excluded as the best treatment for those was deemed to be direct revascularisation without Fractional Flow reserve (FFR) assessment. The diagnostic accuracy of stress CMR was assumed 88% and FFR 100%. A simple cost analysis model was created with the data collected from stress CMR examinations and using the result in an FFR guided strategy. The United Kingdom or United States cost for each investigation used in the model is shown in table 1.

**Table 1:** Cost tariff

	United Kingdom	United States
Stress CMR (Cost per correct diagnosis)	£548 (£623)	$621 ($706)

Coronary angiography	£1052	$2989

CA + FFR	£1512	$3704

PCI	£3676	$6529

Cost of PCI (follow on from FFR)	£2164	$2825

## Results

1,167 patients were included (74% males with a mean age 64 years). Significant MVD was present in 391 patients (33%). 157/391 (40%) underwent stress CMR guided approach. The remaining 234 patients either underwent direct revascularisation (presence of severe or critical stenosis) or underwent a stress echo guided treatment or died in hospital or were lost to follow up. Of those patients undergoing stress CMR, only 39% (61/157) had evidence of inducible myocardial perfusion defect. Putting these figures in a FFR guided approach model our study showed an average saving of £302 per patient or $1558 per patient in a UK based or US based cost assessment, respectively.

## Conclusions

Our study demonstrated that, less than 40% patients undergoing PPCI with moderate to severe by-stander non-culprit coronary artery disease need further revascularisation. As a gatekeeper to complete revascularisation, stress CMR was also found to be a cheaper management strategy in a cost analysis model when UK or US-based costs were assumed.

## Funding

This study was funded by the National Institute for Health Research Biomedical Research Unit in Cardiovascular Disease at the University Hospitals Bristol NHS Foundation Trust and the University of Bristol.

